# 681. Penicillin Plus Ceftriaxone Against *Enterococcus faecalis* In Vitro Time-Kill Studies

**DOI:** 10.1093/ofid/ofab466.878

**Published:** 2021-12-04

**Authors:** Ruhmazia Khan, Zeel Shah, Vanthida Huang, Jaclyn Cusumano

**Affiliations:** 1 Long Island University, Brooklyn, New York; 2 Midwestern University College of Pharmacy - Glendale, Glendale, Arizona; 3 Mount Sinai Queens Hospital, Queens, New York

## Abstract

**Background:**

Synergistic ampicillin plus ceftriaxone (AC) for *Enterococcus faecalis* infective endocarditis outpatient use is precluded by ampicillin’s poor room temperature stability. Penicillin has superior stability and has been combined with ceftriaxone (PC), however there is a lack of studies to demonstrate synergy.

**Methods:**

AC and PC were evaluated, in duplicate, for synergy utilizing 24-hour in vitro time-kill assays with a starting inoculum of 10^6^ colony forming units (CFU)/mL. Six clinical *E. faecalis* blood isolates and one wild-type *E. faecalis* isolate (JH2-2) were included. All isolates were susceptible to ampicillin and penicillin, with minimum inhibitory concentrations (MICs) ranging from 0.5-1 µg/mL and 2-4 µg/mL, respectively. Ampicillin and penicillin were tested at subinhibitory concentrations (0.25x and 0.5xMIC) as monotherapy and in combination with ceftriaxone average steady state concentrations for a dose of 2g IV q12hr (*CPss* 17.2 µg/mL), as all ceftriaxone MICs were high due to intrinsic resistance (MICs 128-2048 µg/mL). Synergy was defined as a ≥ 2 log_10_ decrease in CFU/mL at 24 hours from the most active single agent.

**Results:**

An average increase in bacterial density from the starting inoculum was observed for all isolates against ampicillin 0.25xMIC alone, penicillin 0.25x and 0.5xMIC alone, and ceftriaxone alone (+1.60 ± 0.62, +1.91 ± 0.37, +1.48 ± 0.42, and +1.84 ± 0.46 log_10_ CFU/mL, respectively) [Table 1]. Ampicillin 0.5xMIC alone average increase in bacterial density from starting inoculum for all but two isolates (e2008 and e2009) was +1.21 ± 0.59 log_10_ CFU/mL. Isolates e2008 and e2009 were the only isolates with a higher penicillin MIC of 4 µg/mL, and did not display synergy for all AC and all PC combinations. AC synergy was observed for all other isolates, with only one isolate (e2012) displaying synergy at 0.5xMIC. PC synergy was observed for four isolates at 0.5xMIC (-3.47 ± 0.94 log_10_ CFU/mL) and for only one isolate (e2014) at 0.25xMIC but the change in bacterial density was -0.38 ± 0.24 log_10_ CFU/mL.

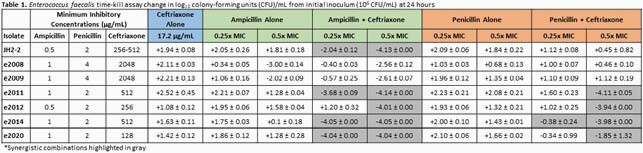

**Conclusion:**

PC synergy against *E. faecalis* was observed with higher penicillin concentrations. AC and PC did not demonstrate synergy against isolates with a higher penicillin MIC of 4 µg/mL. Further research is warranted to better understand PC synergy against *E. faecalis*.

**Disclosures:**

**All Authors**: No reported disclosures

